# Supplementary figures and images for: Signaling pathways as linear transmitters
Source: eLife. 2018 Sep 19;7:e33617. doi: 10.7554/eLife.33617 (PMC6202053; doi:10.7554/eLife.33617)

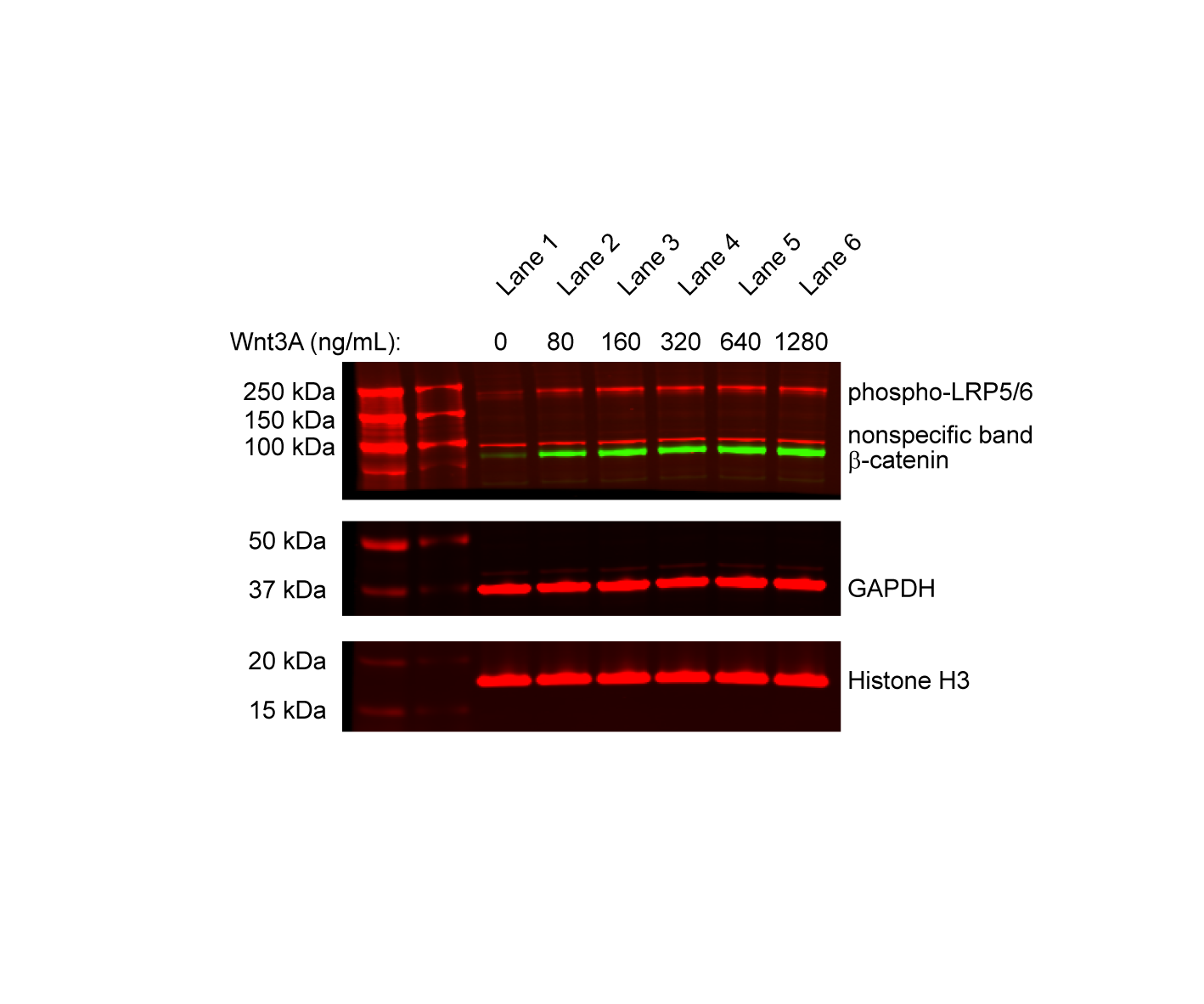

Supplement: Figure 3—source data 1. [file elife-33617-fig3-data1.zip › Figure 3 Source Data 1/Figure 3A Gel Images/Figure 3A - Gel 10-01.tif]

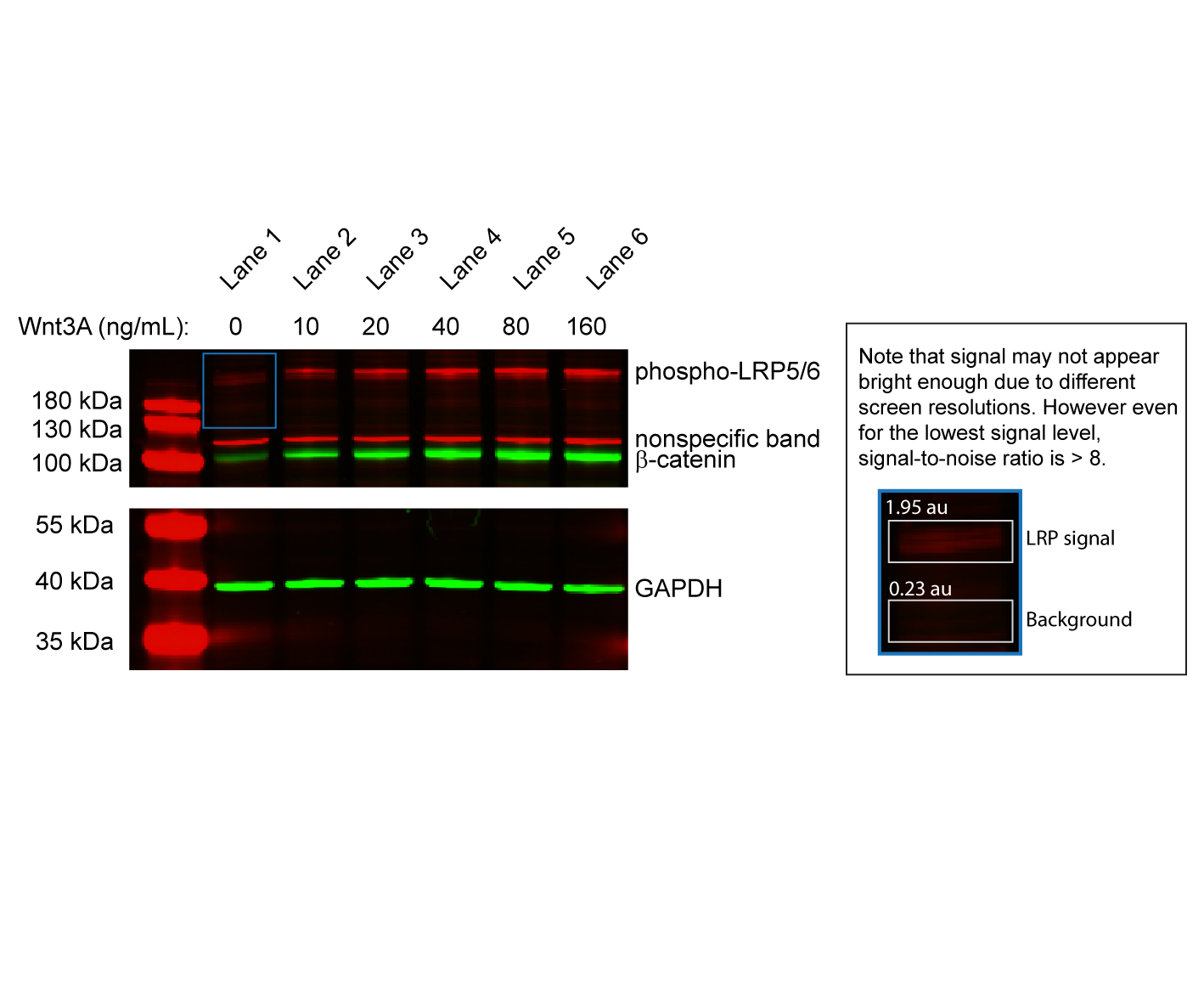

Supplement: Figure 3—source data 1. [file elife-33617-fig3-data1.zip › Figure 3 Source Data 1/Figure 3A Gel Images/Figure 3A - Gel 1-01.tif]

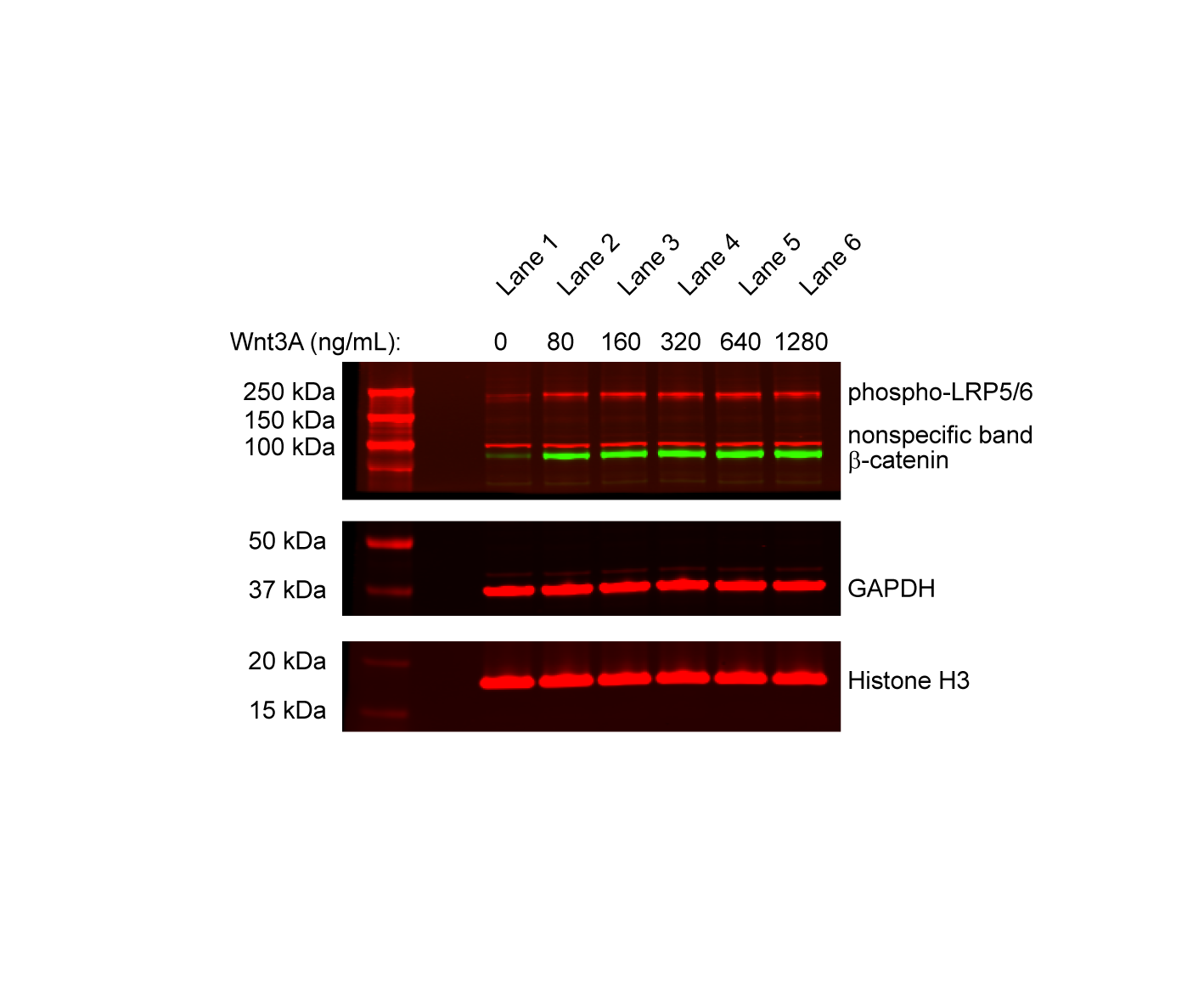

Supplement: Figure 3—source data 1. [file elife-33617-fig3-data1.zip › Figure 3 Source Data 1/Figure 3A Gel Images/Figure 3A - Gel 11-01.tif]

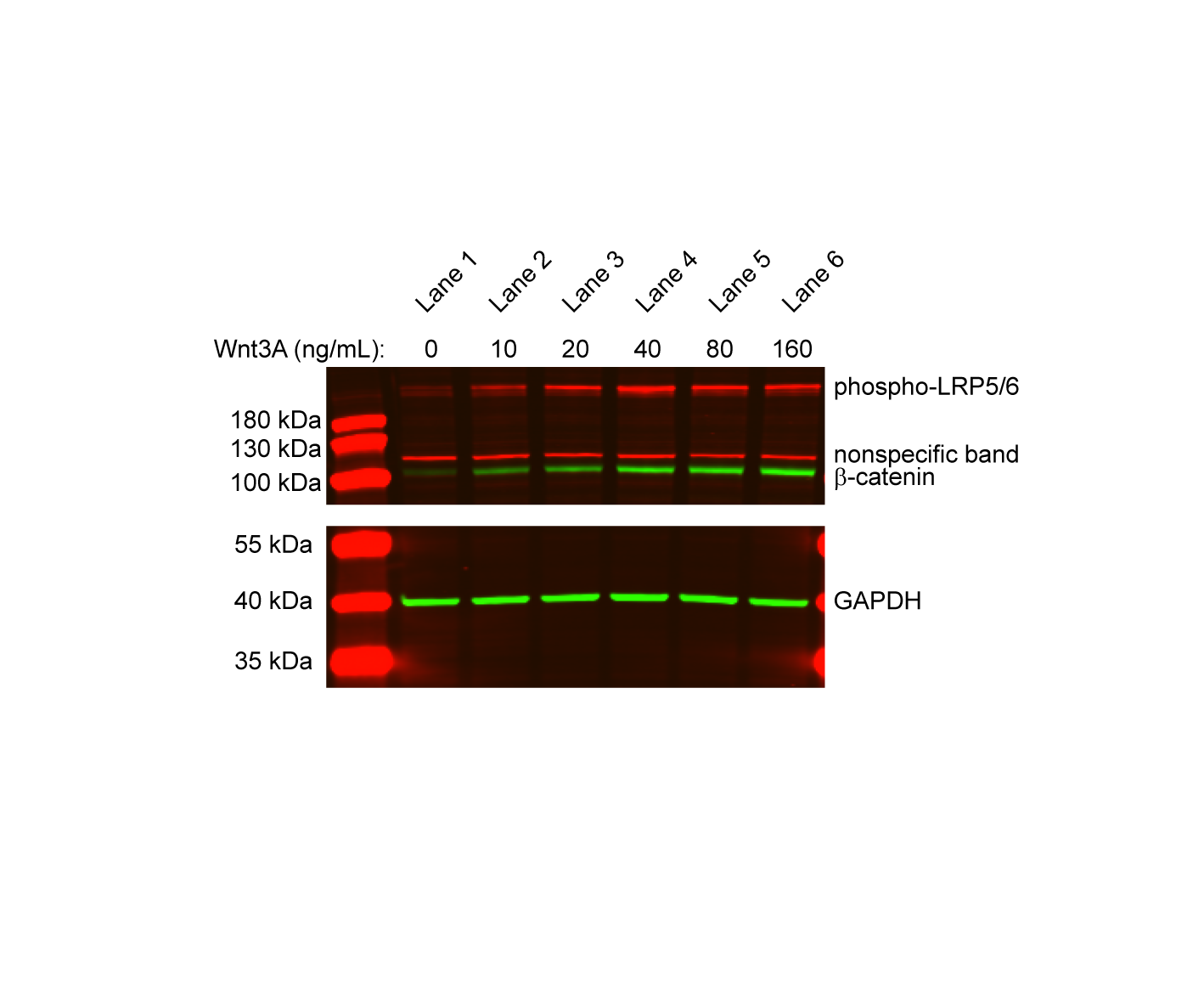

Supplement: Figure 3—source data 1. [file elife-33617-fig3-data1.zip › Figure 3 Source Data 1/Figure 3A Gel Images/Figure 3A - Gel 2-01.tif]

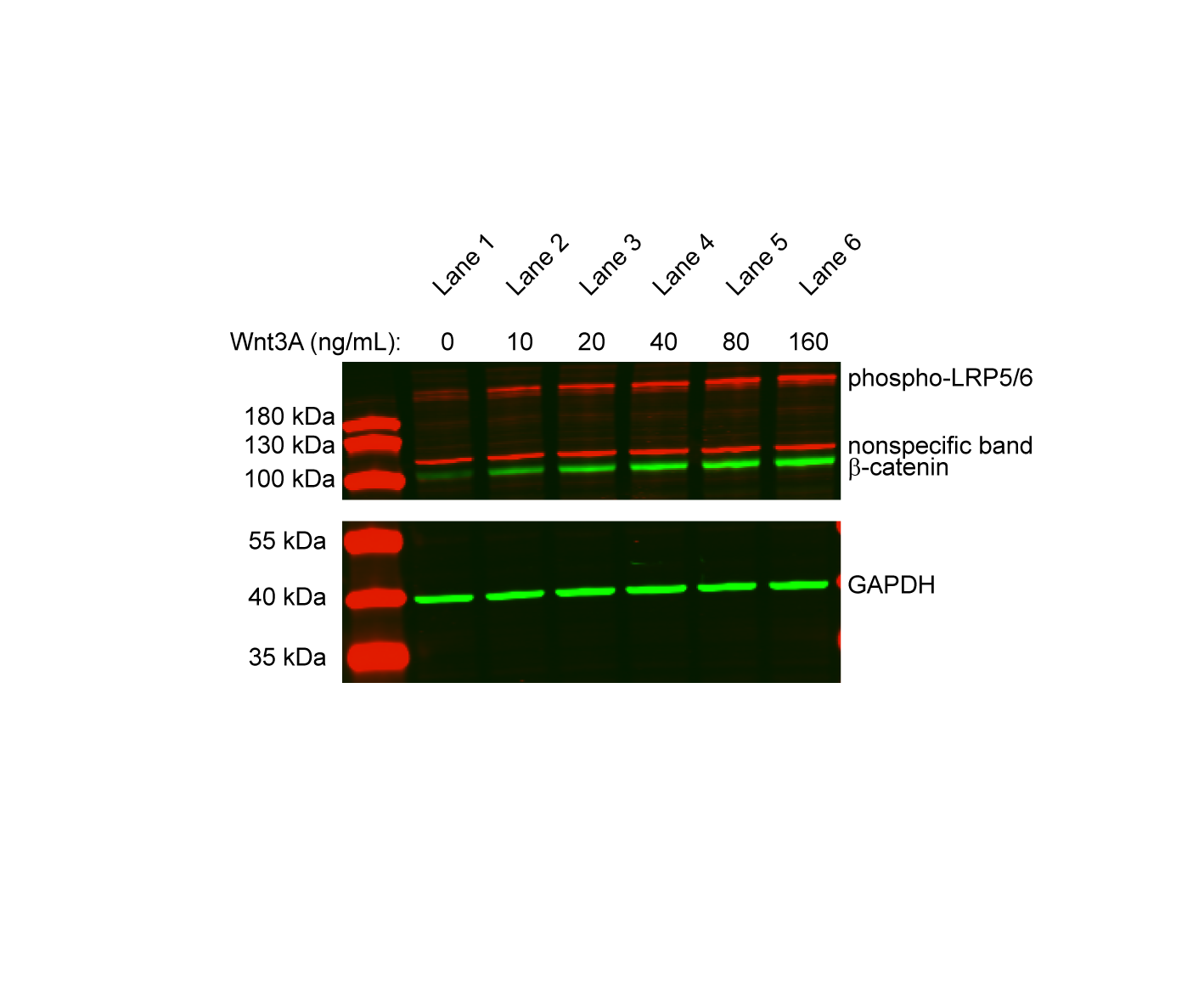

Supplement: Figure 3—source data 1. [file elife-33617-fig3-data1.zip › Figure 3 Source Data 1/Figure 3A Gel Images/Figure 3A - Gel 3-01.tif]

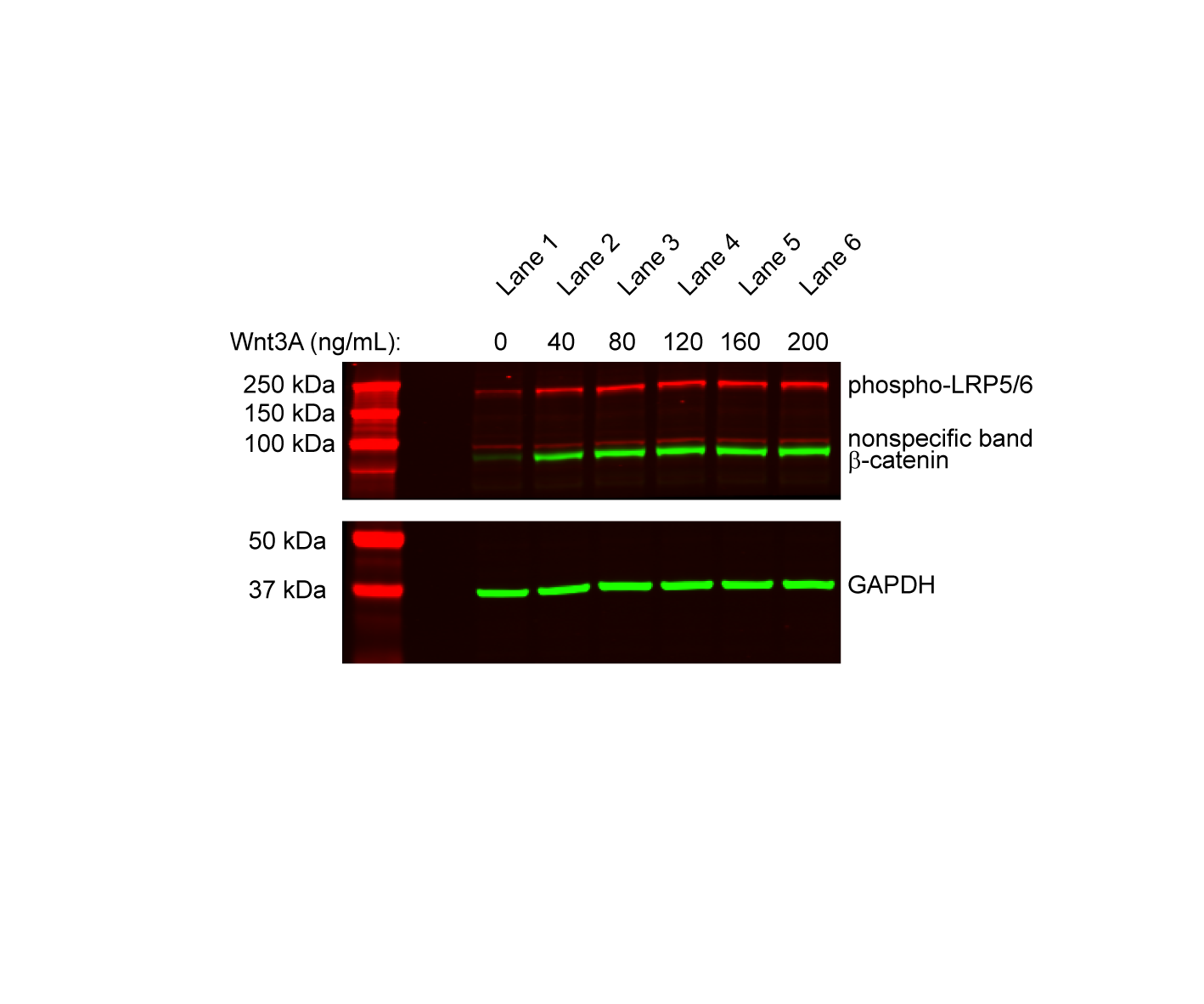

Supplement: Figure 3—source data 1. [file elife-33617-fig3-data1.zip › Figure 3 Source Data 1/Figure 3A Gel Images/Figure 3A - Gel 4-01.tif]

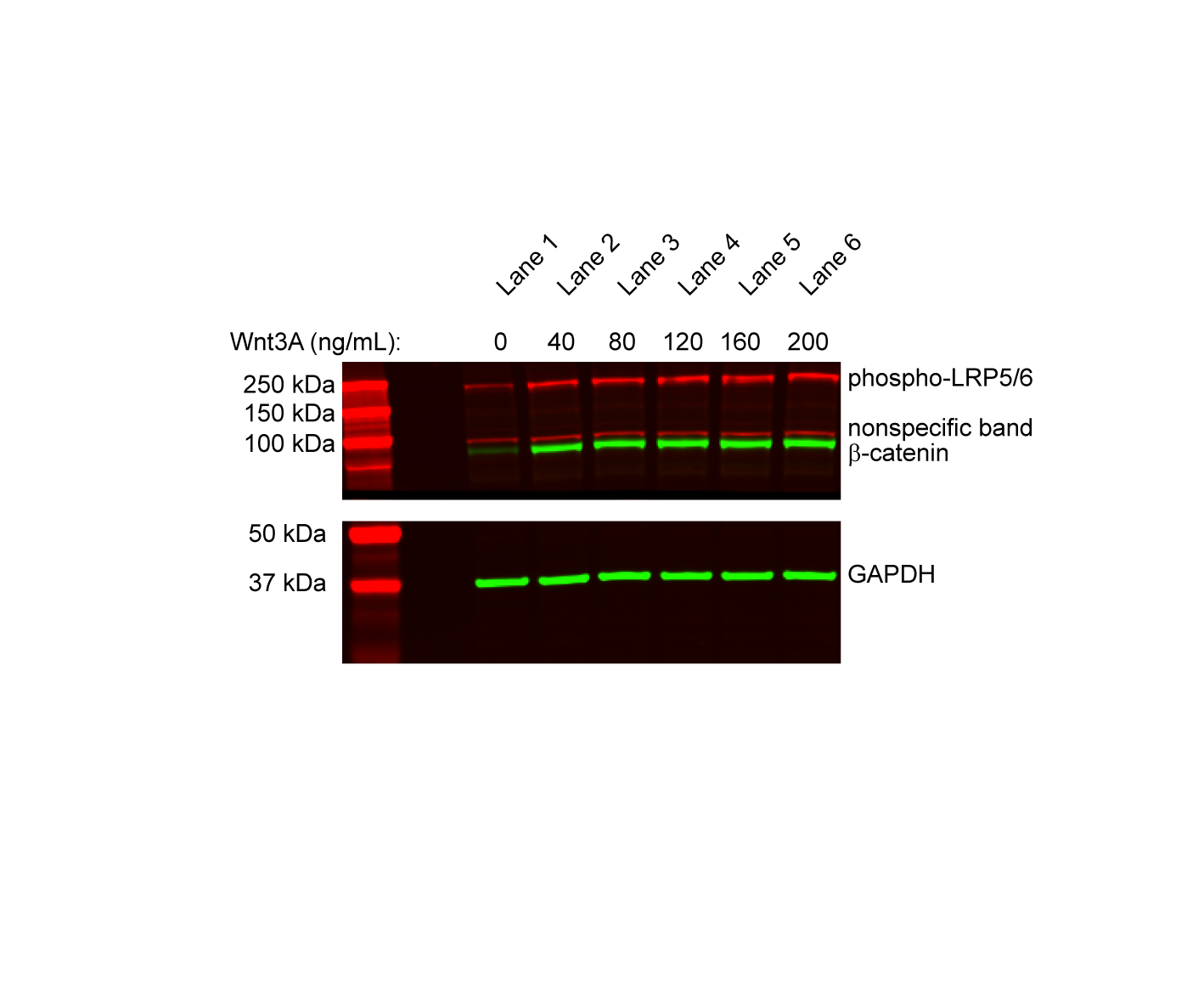

Supplement: Figure 3—source data 1. [file elife-33617-fig3-data1.zip › Figure 3 Source Data 1/Figure 3A Gel Images/Figure 3A - Gel 5-01.tif]

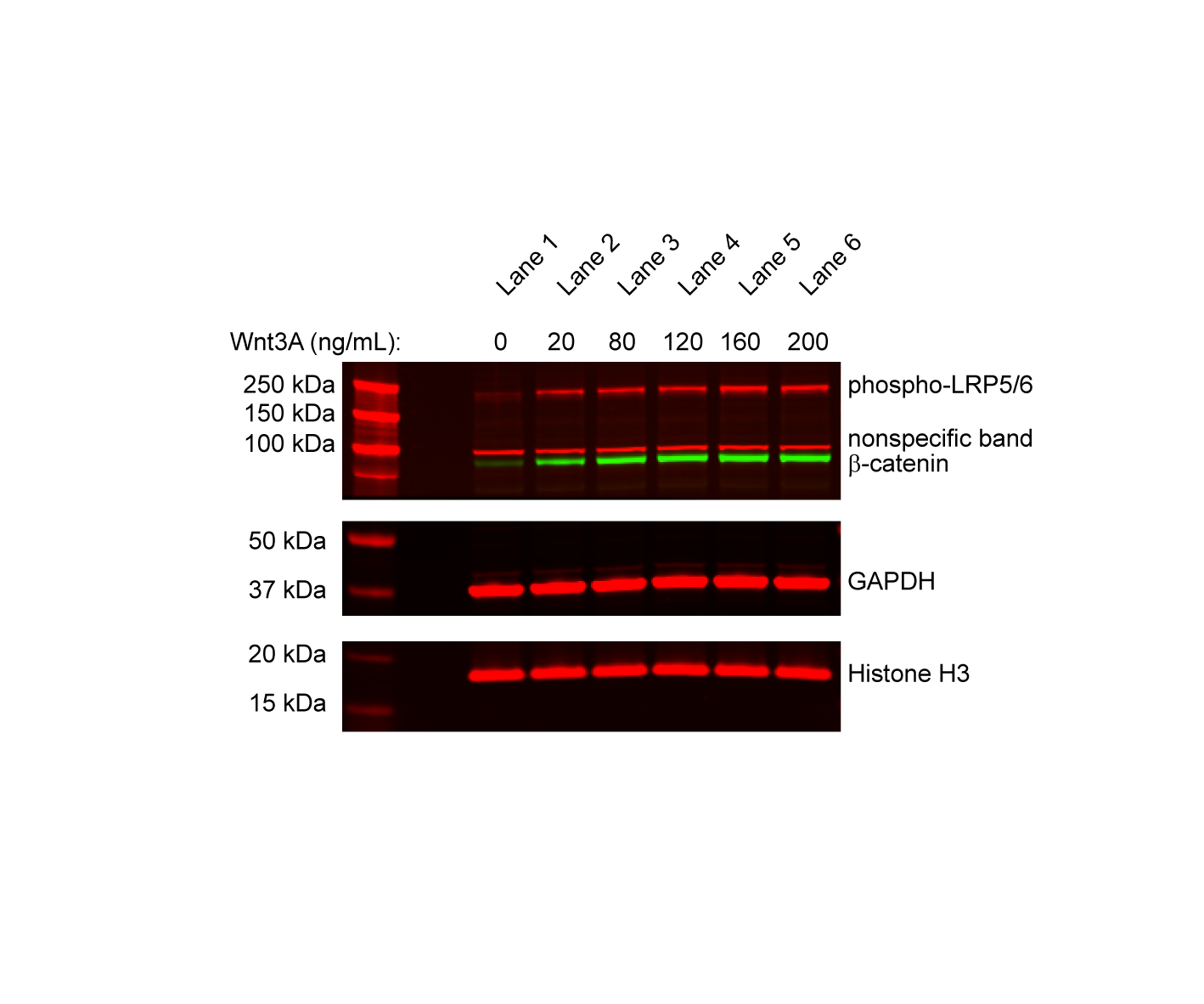

Supplement: Figure 3—source data 1. [file elife-33617-fig3-data1.zip › Figure 3 Source Data 1/Figure 3A Gel Images/Figure 3A - Gel 6-01.tif]

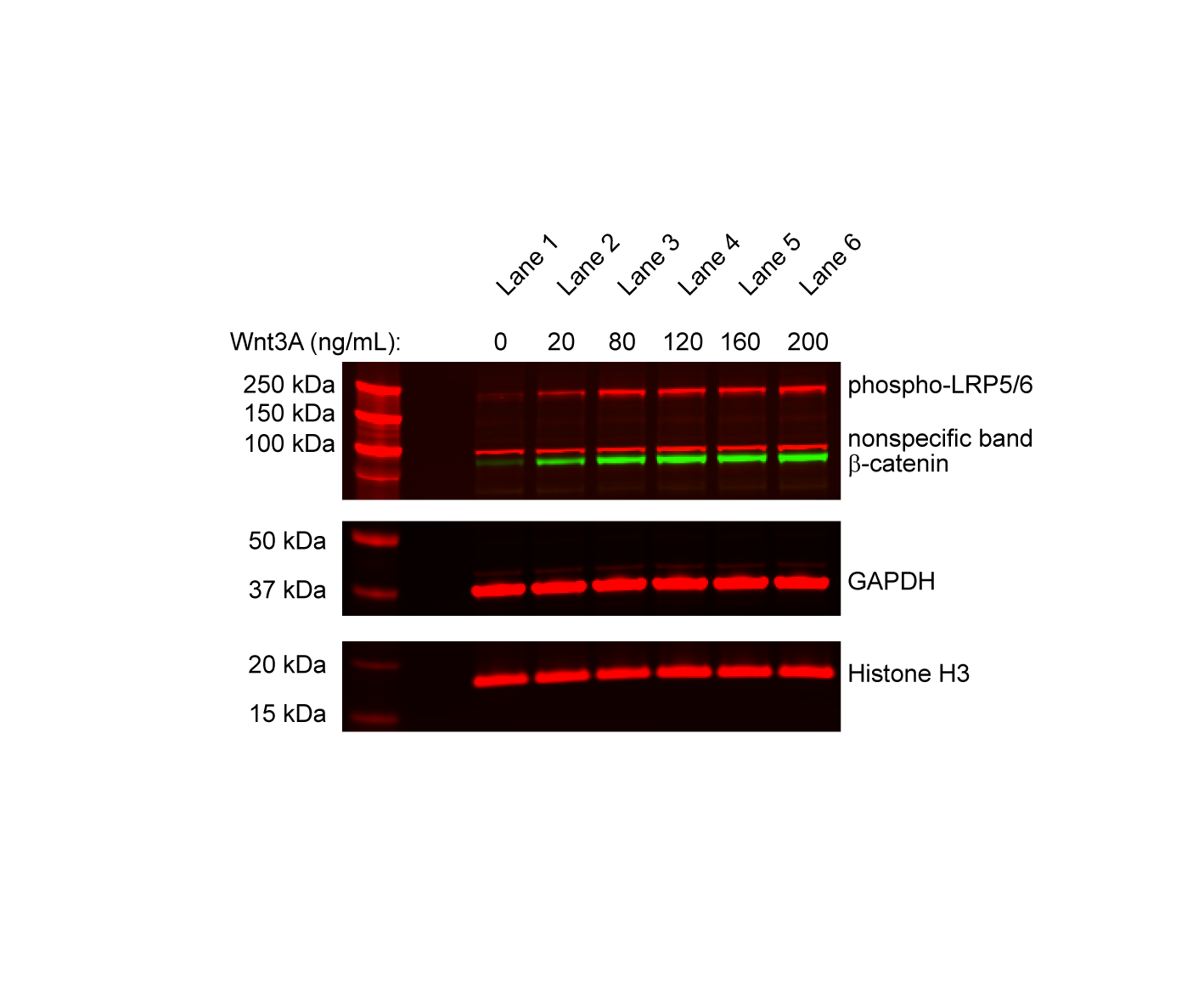

Supplement: Figure 3—source data 1. [file elife-33617-fig3-data1.zip › Figure 3 Source Data 1/Figure 3A Gel Images/Figure 3A - Gel 7-01.tif]

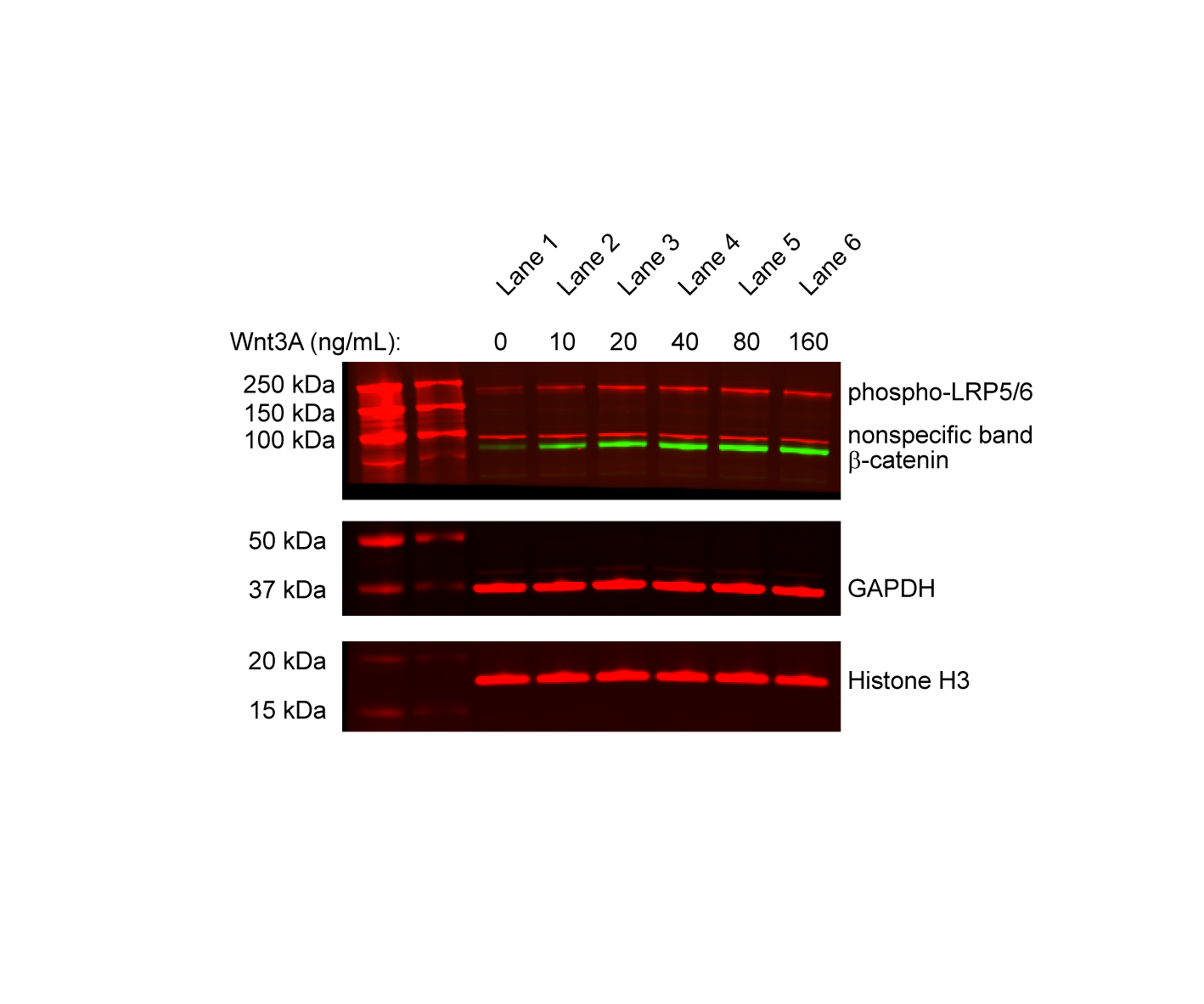

Supplement: Figure 3—source data 1. [file elife-33617-fig3-data1.zip › Figure 3 Source Data 1/Figure 3A Gel Images/Figure 3A - Gel 8-01.tif]

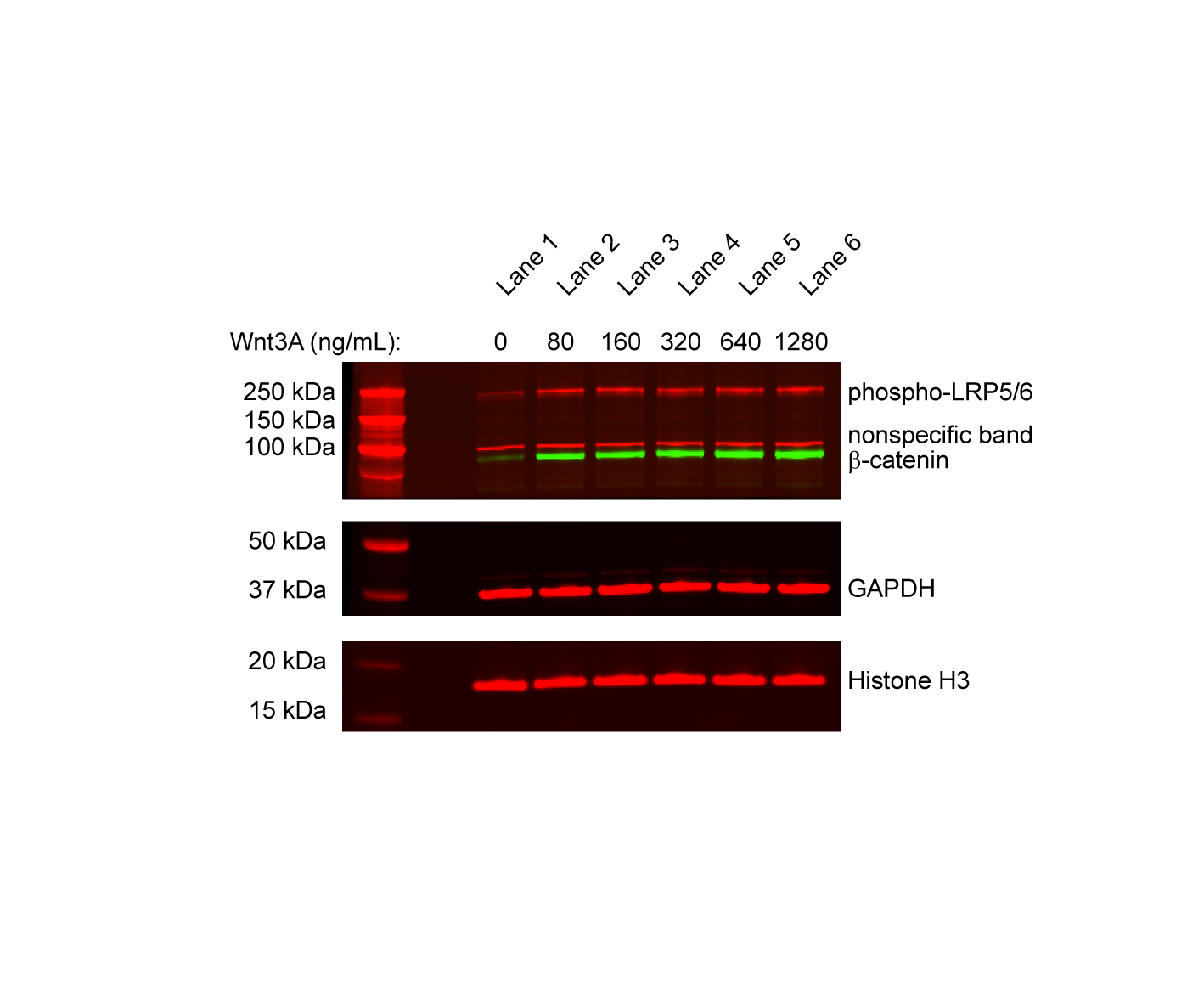

Supplement: Figure 3—source data 1. [file elife-33617-fig3-data1.zip › Figure 3 Source Data 1/Figure 3A Gel Images/Figure 3A - Gel 9-01.tif]

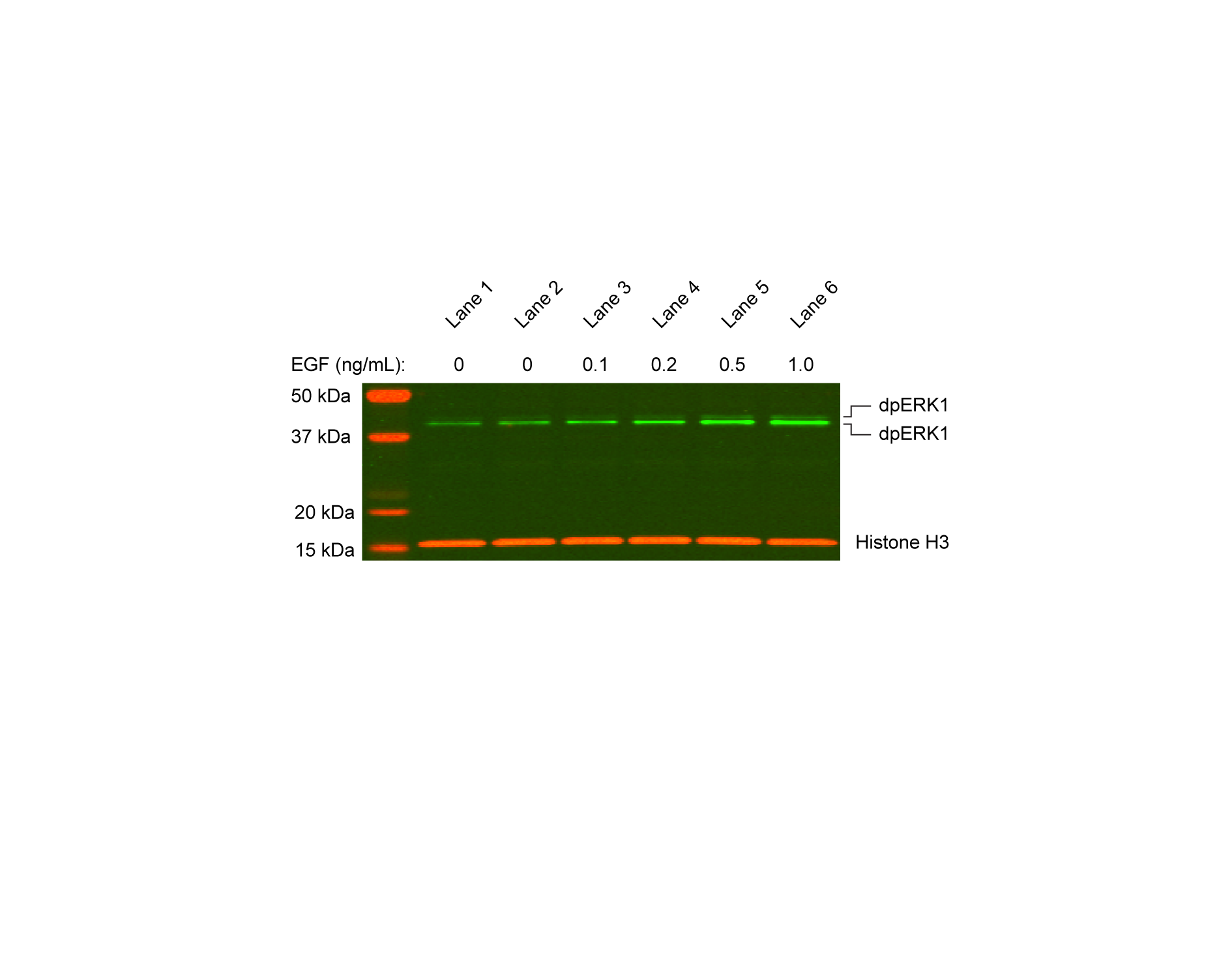

Supplement: Figure 3—source data 1. [file elife-33617-fig3-data1.zip › Figure 3 Source Data 1/Figure 3B Gel Images/Figure 3B - Gel 1-01.tif]

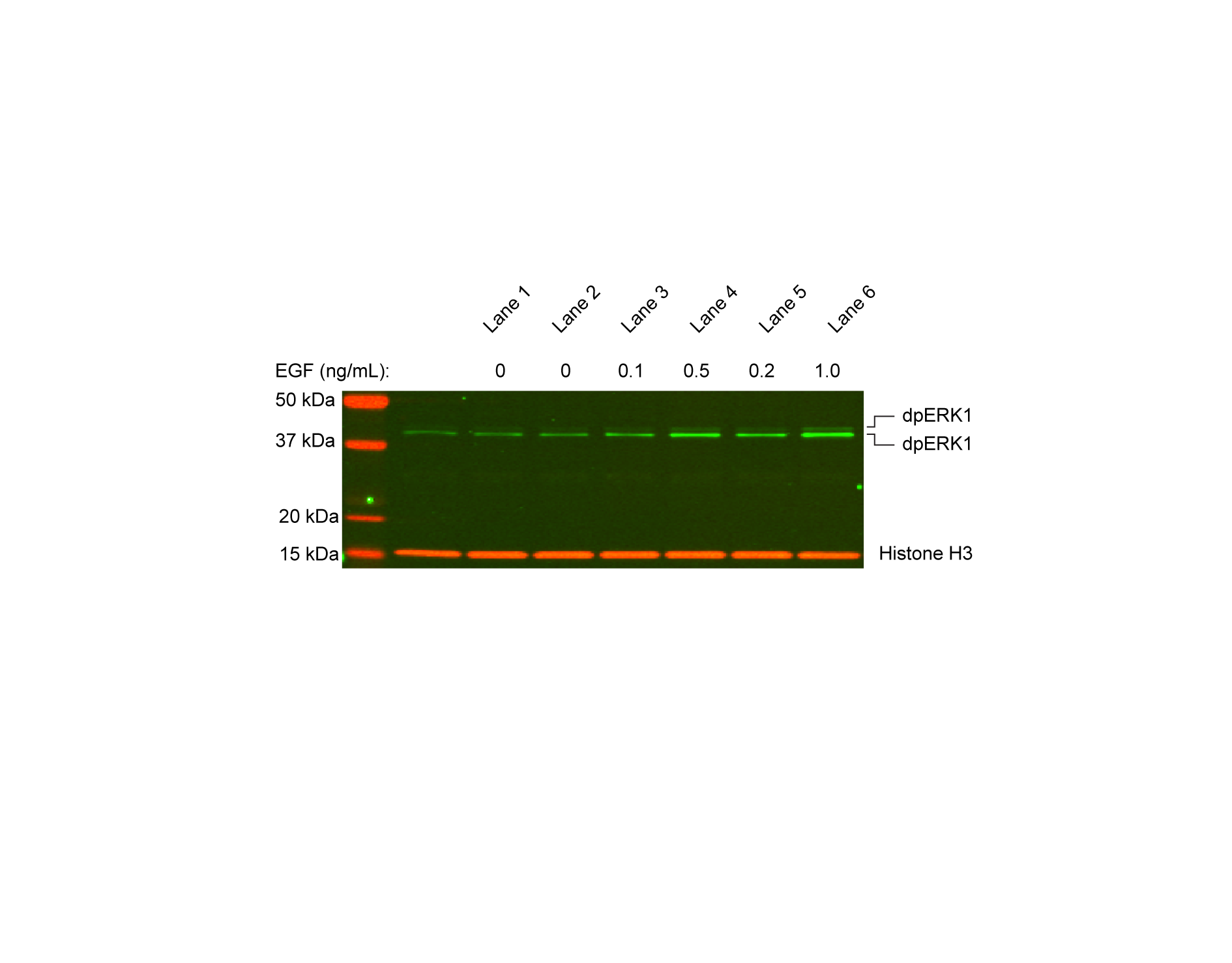

Supplement: Figure 3—source data 1. [file elife-33617-fig3-data1.zip › Figure 3 Source Data 1/Figure 3B Gel Images/Figure 3B - Gel 2-01.tif]

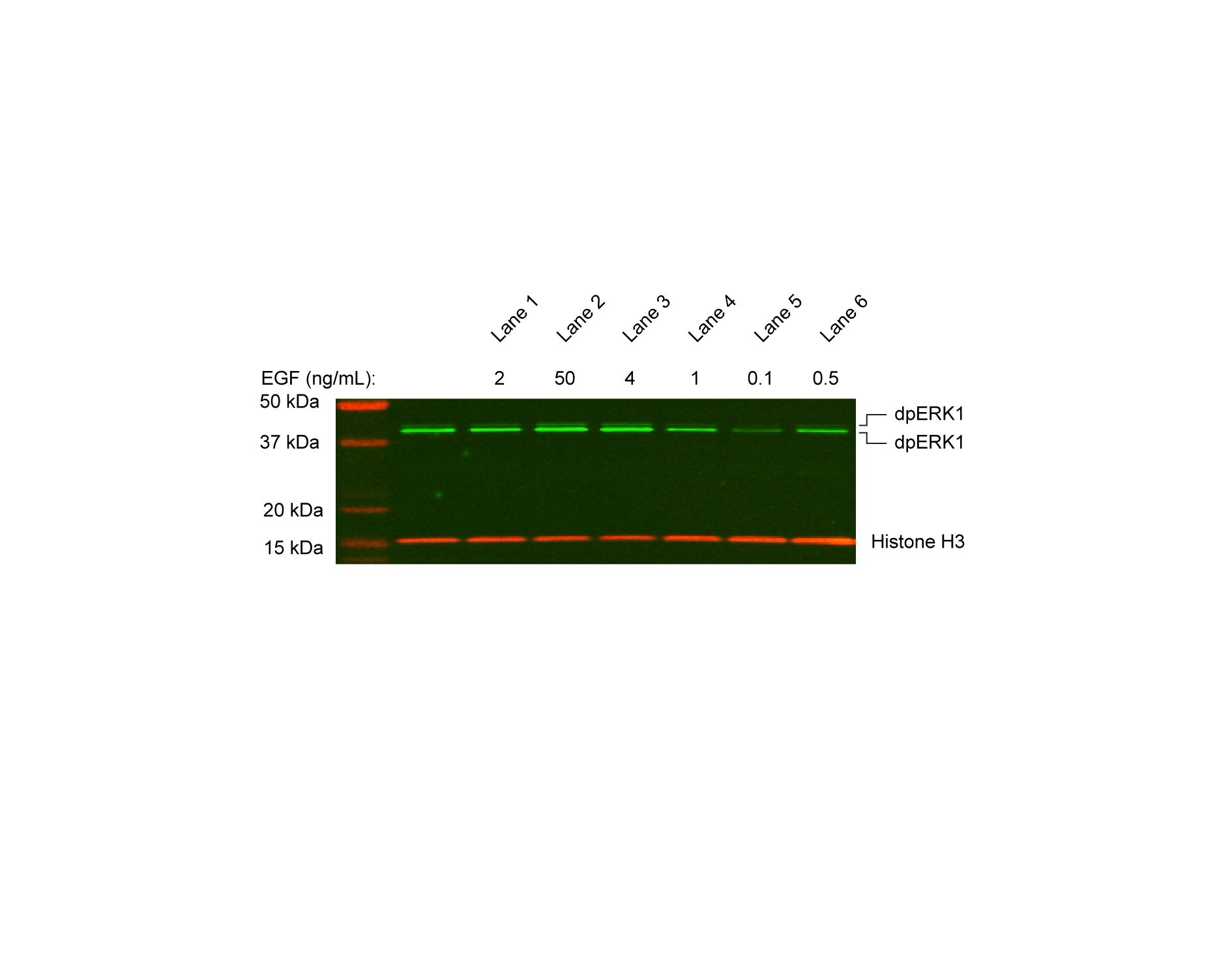

Supplement: Figure 3—source data 1. [file elife-33617-fig3-data1.zip › Figure 3 Source Data 1/Figure 3B Gel Images/Figure 3B - Gel 3-01.tif]

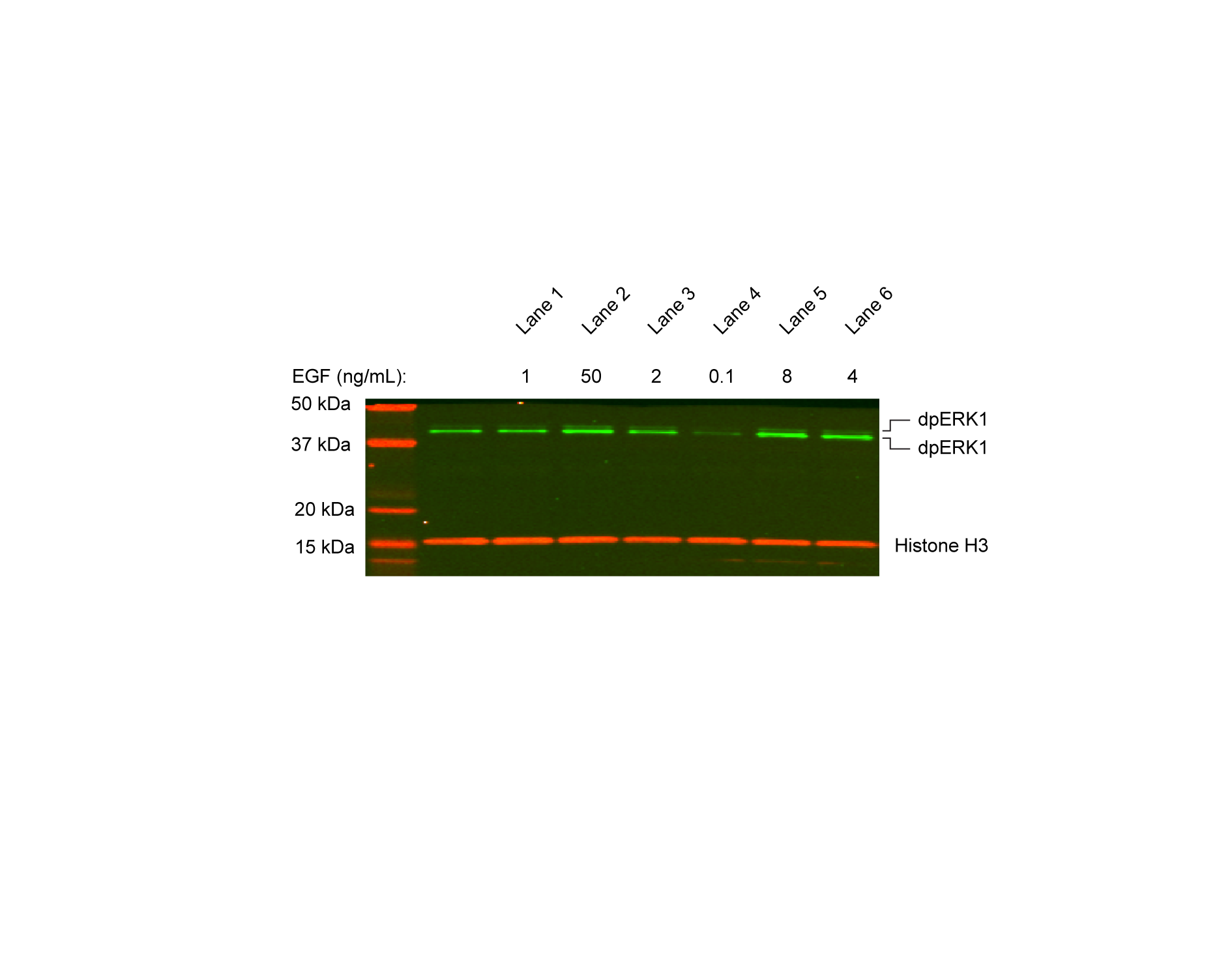

Supplement: Figure 3—source data 1. [file elife-33617-fig3-data1.zip › Figure 3 Source Data 1/Figure 3B Gel Images/Figure 3B - Gel 4-01.tif]

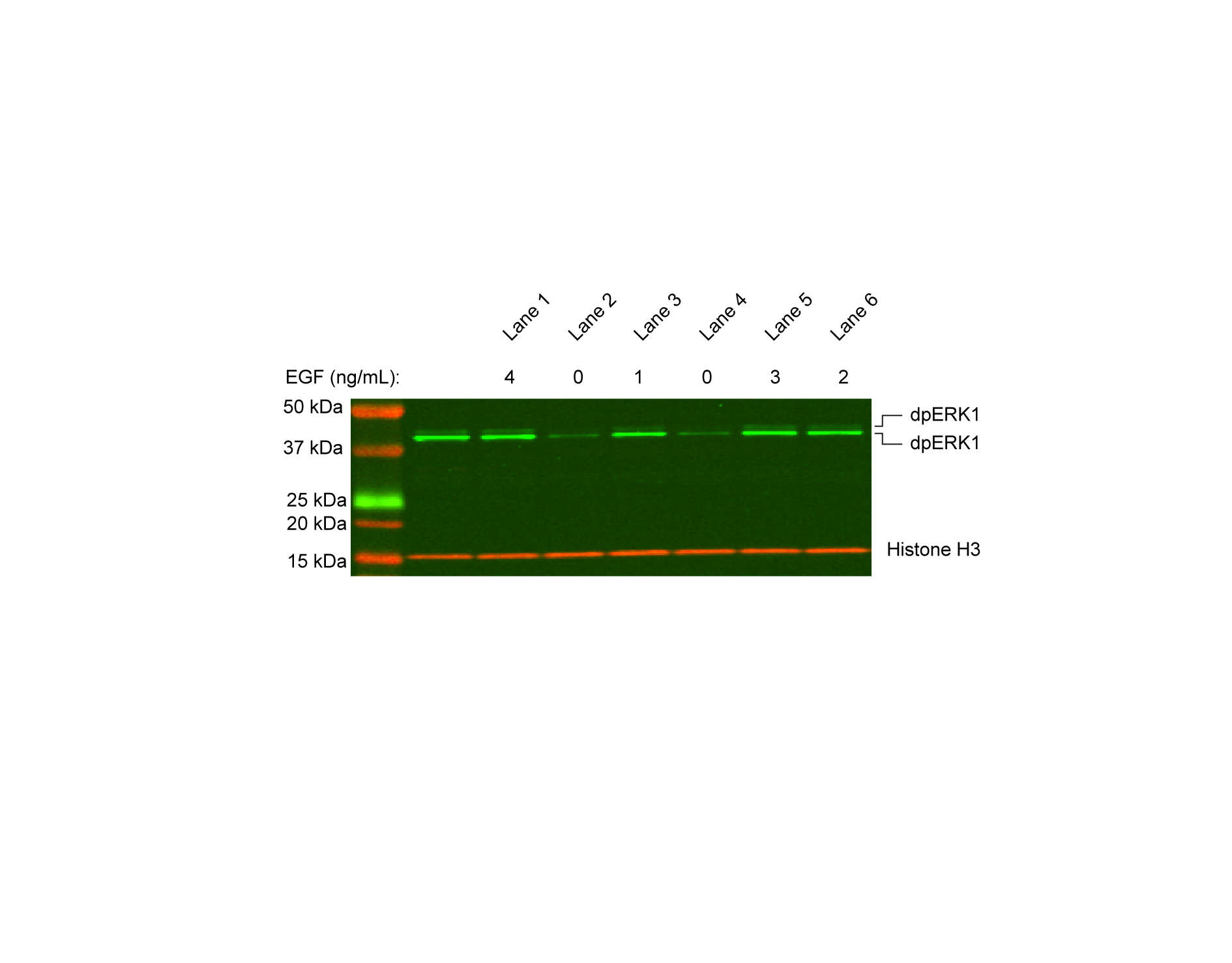

Supplement: Figure 3—source data 1. [file elife-33617-fig3-data1.zip › Figure 3 Source Data 1/Figure 3B Gel Images/Figure 3B - Gel 5-01.tif]

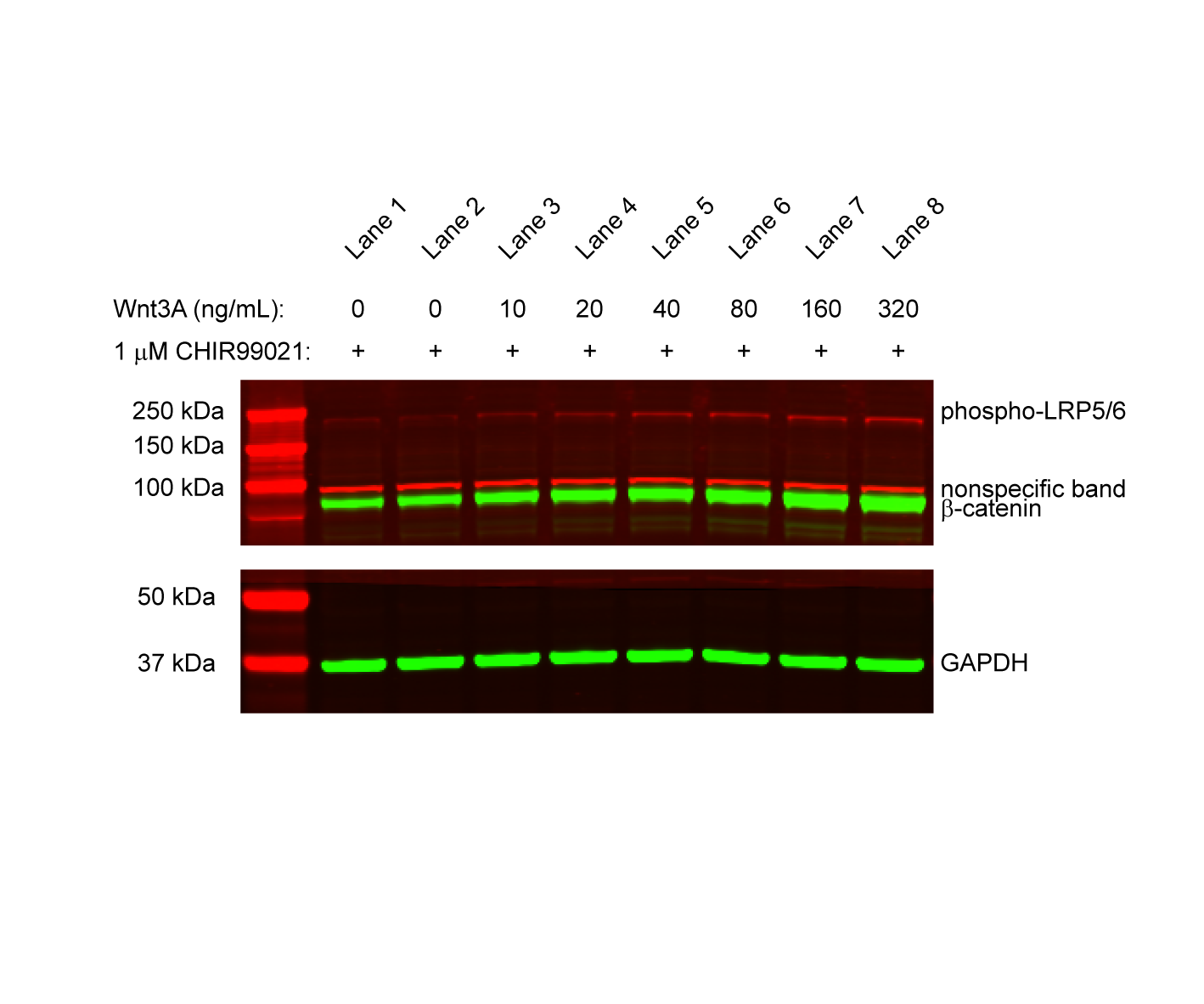

Supplement: Figure 3—source data 1. [file elife-33617-fig3-data1.zip › Figure 3 Source Data 1/Figure 3C Gel Images/Figure 3C - Gel 1-01.tif]

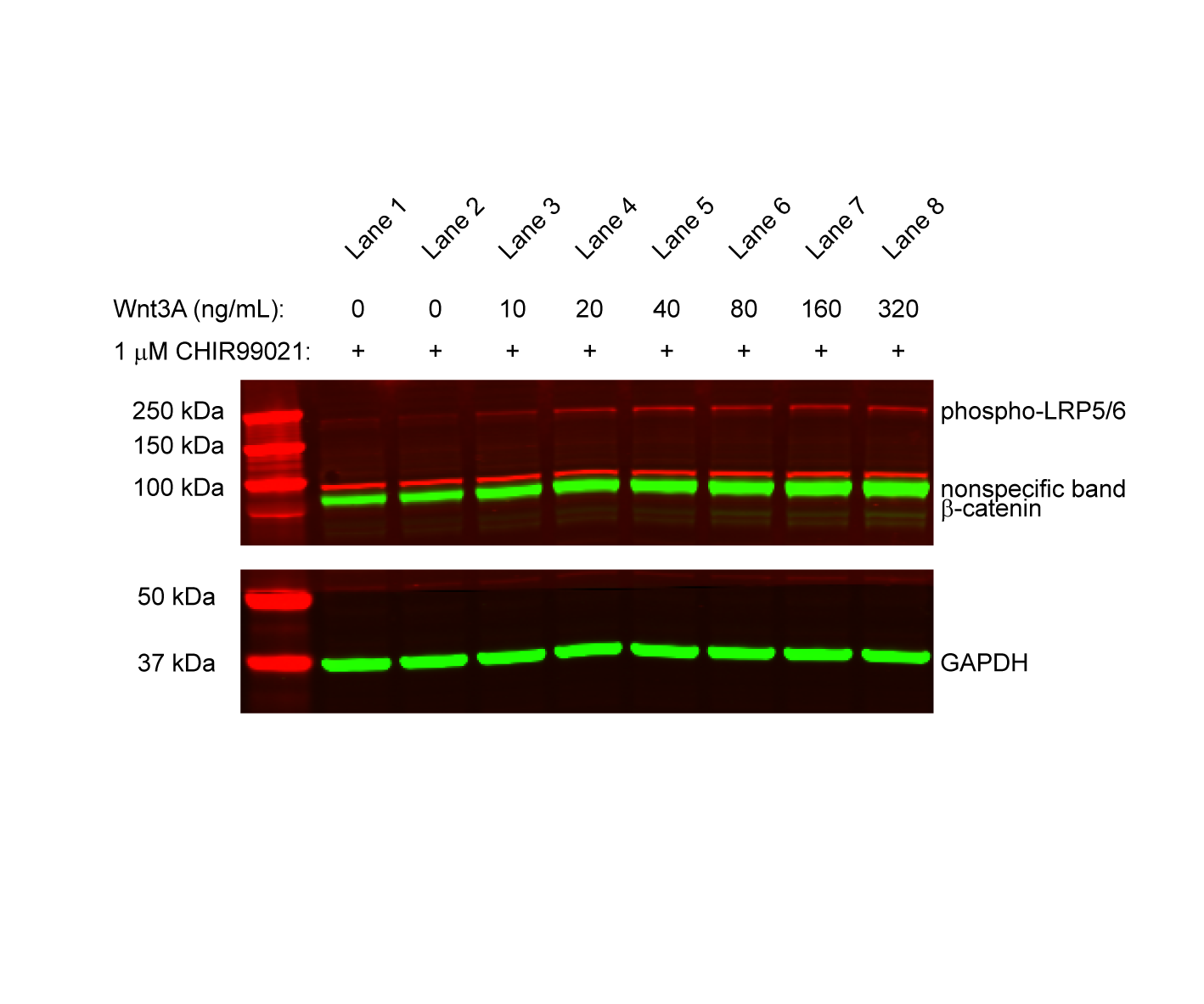

Supplement: Figure 3—source data 1. [file elife-33617-fig3-data1.zip › Figure 3 Source Data 1/Figure 3C Gel Images/Figure 3C - Gel 2-01.tif]

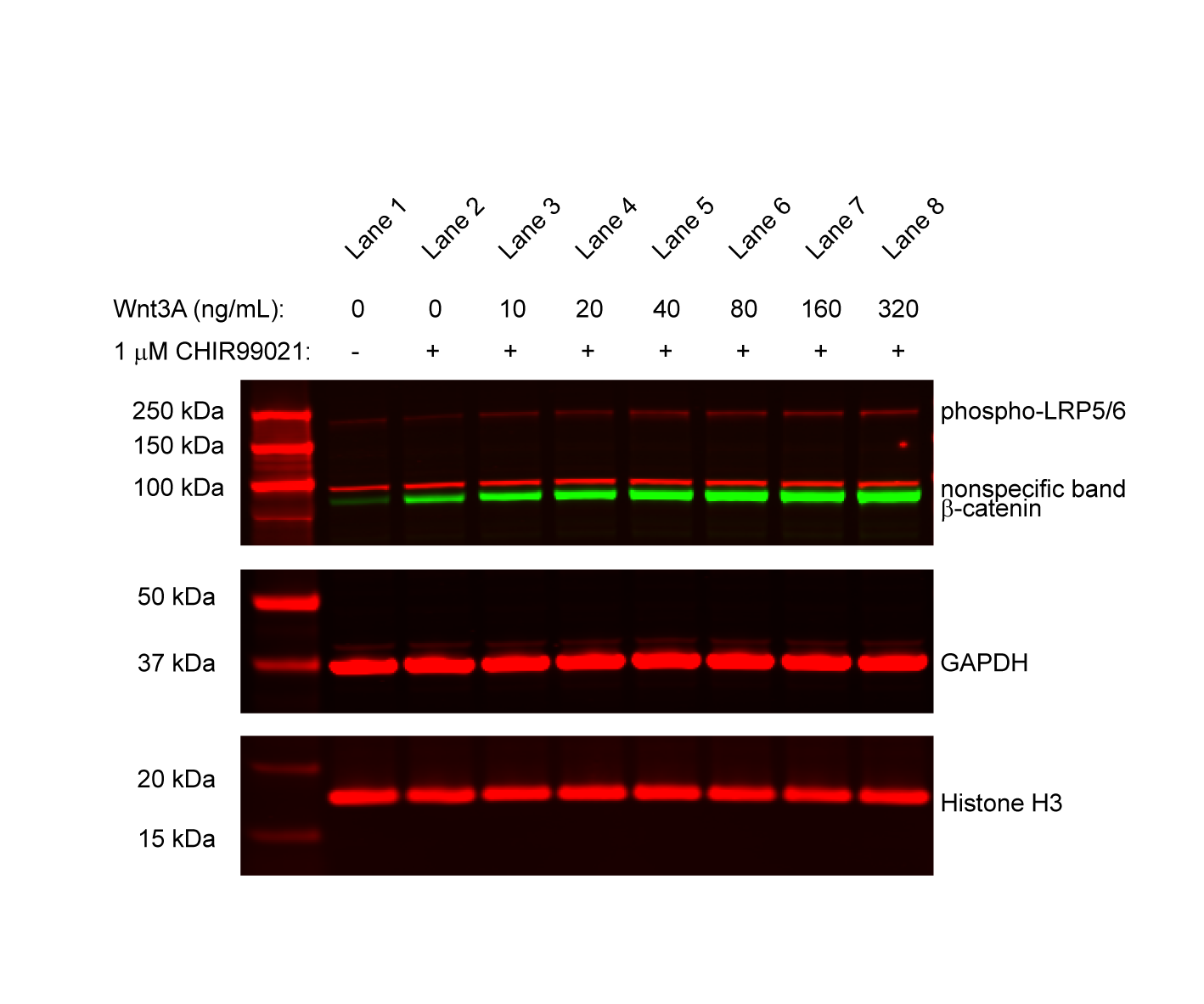

Supplement: Figure 3—source data 1. [file elife-33617-fig3-data1.zip › Figure 3 Source Data 1/Figure 3C Gel Images/Figure 3C - Gel 3-01.tif]

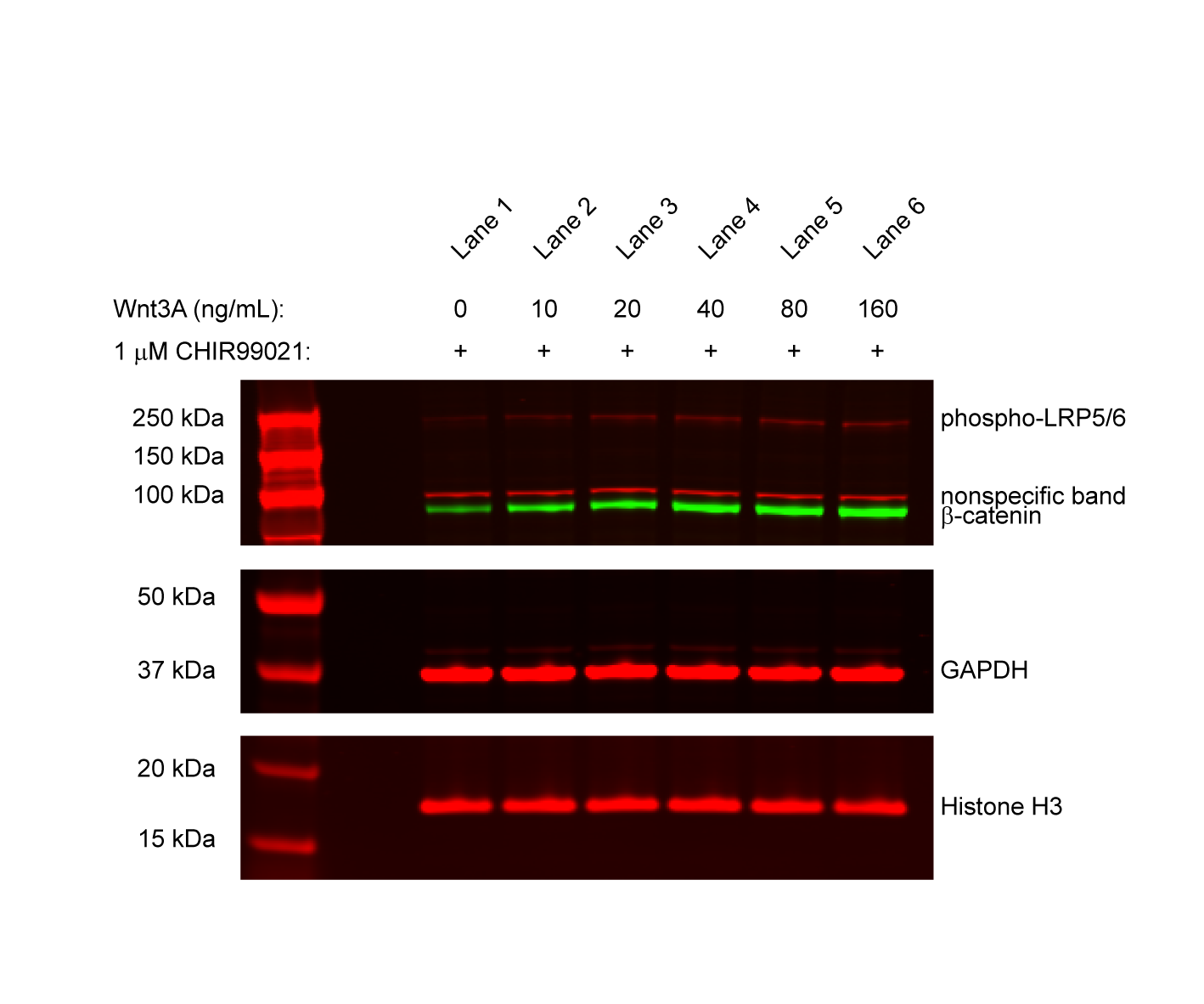

Supplement: Figure 3—source data 1. [file elife-33617-fig3-data1.zip › Figure 3 Source Data 1/Figure 3C Gel Images/Figure 3C - Gel 4-01.tif]

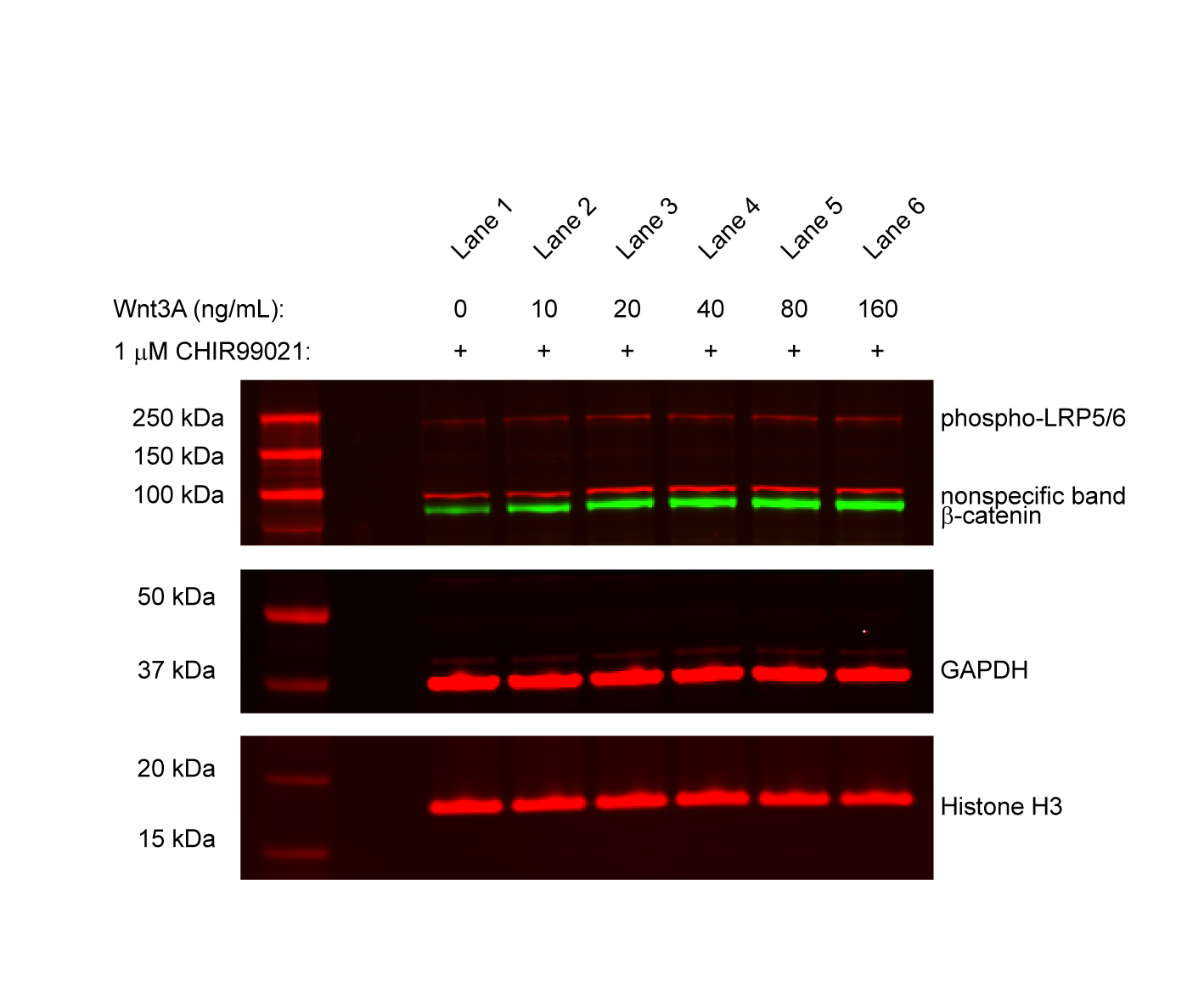

Supplement: Figure 3—source data 1. [file elife-33617-fig3-data1.zip › Figure 3 Source Data 1/Figure 3C Gel Images/Figure 3C - Gel 5-01.tif]

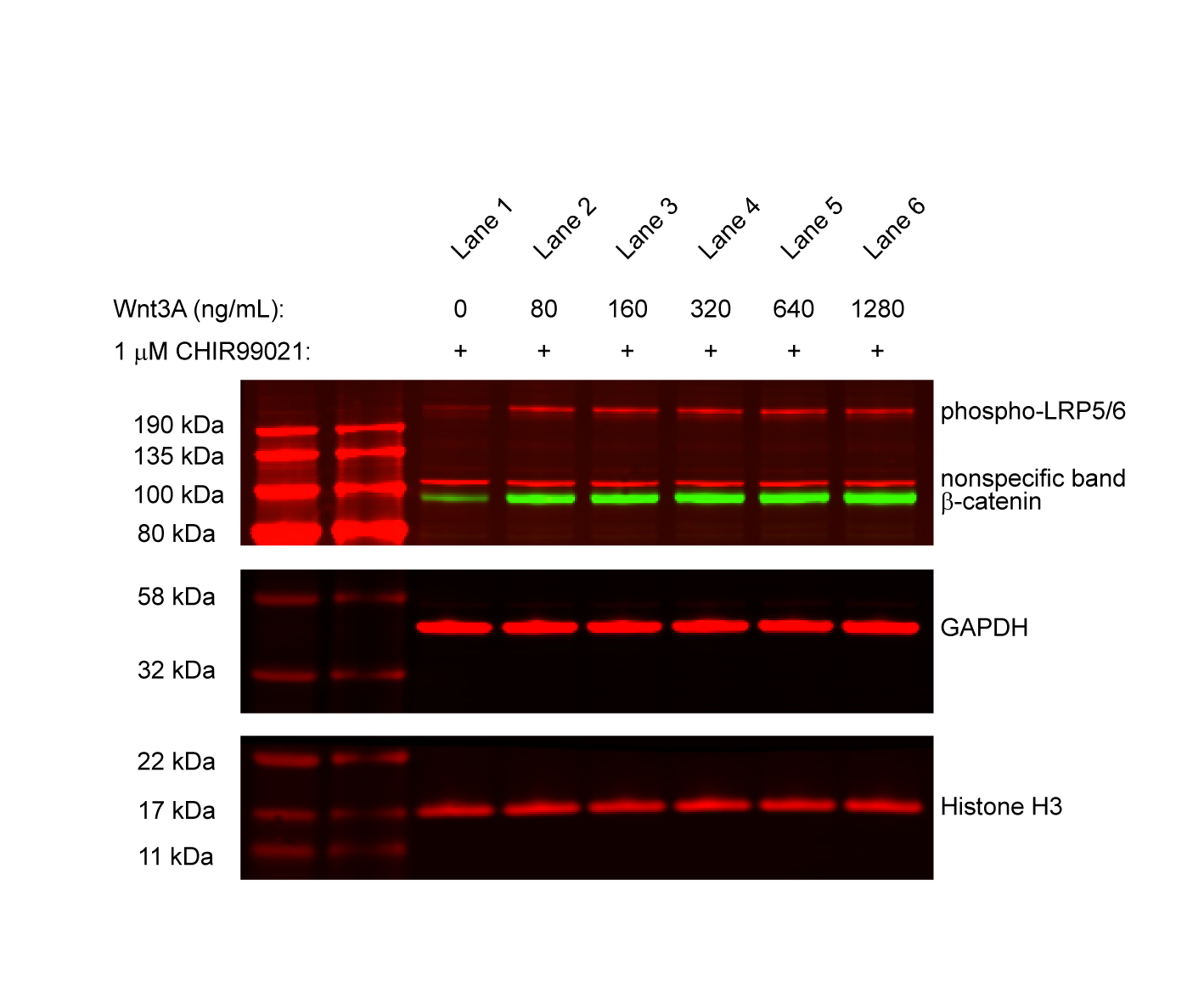

Supplement: Figure 3—source data 1. [file elife-33617-fig3-data1.zip › Figure 3 Source Data 1/Figure 3C Gel Images/Figure 3C - Gel 6-01.tif]

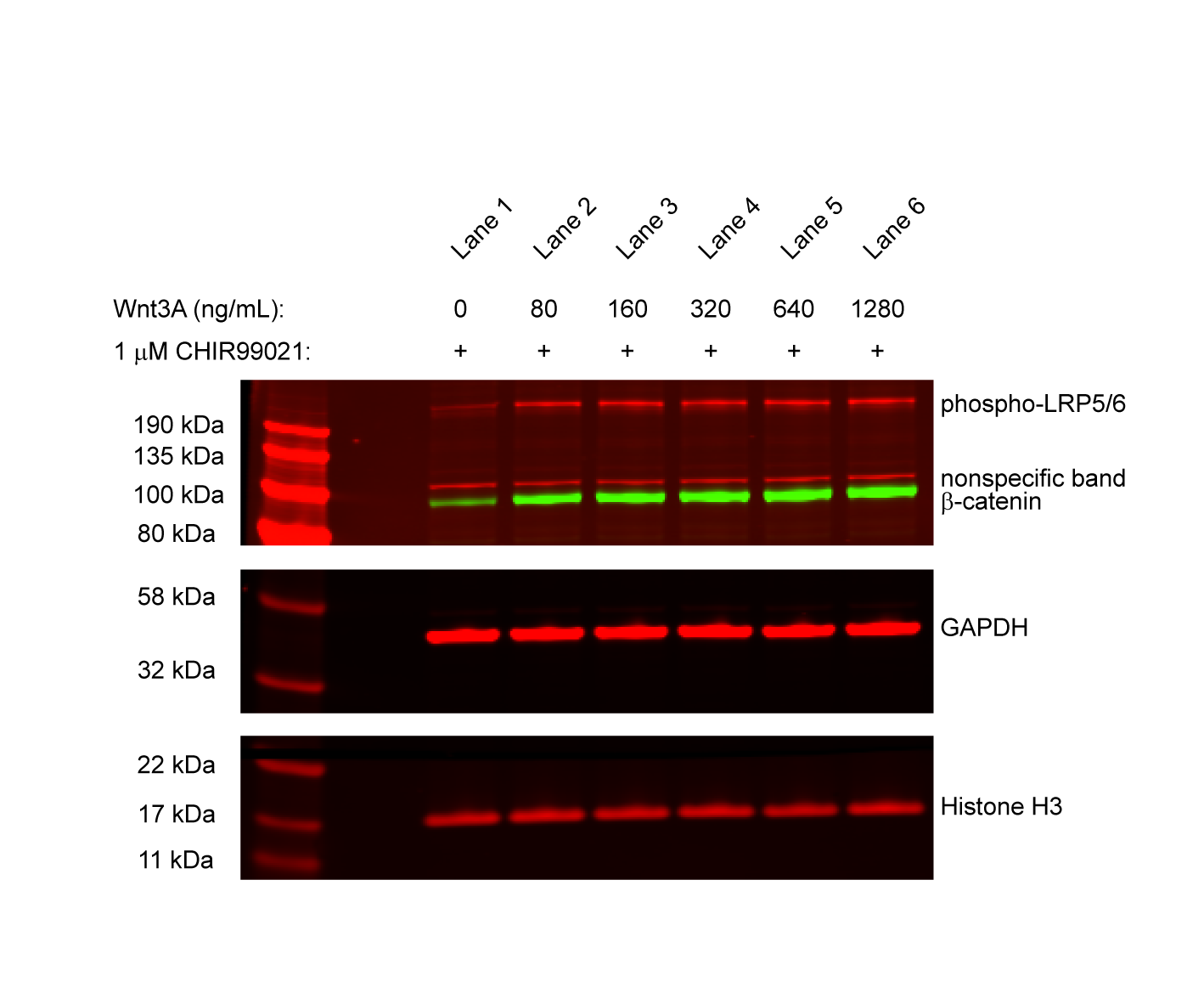

Supplement: Figure 3—source data 1. [file elife-33617-fig3-data1.zip › Figure 3 Source Data 1/Figure 3C Gel Images/Figure 3C - Gel 7-01.tif]

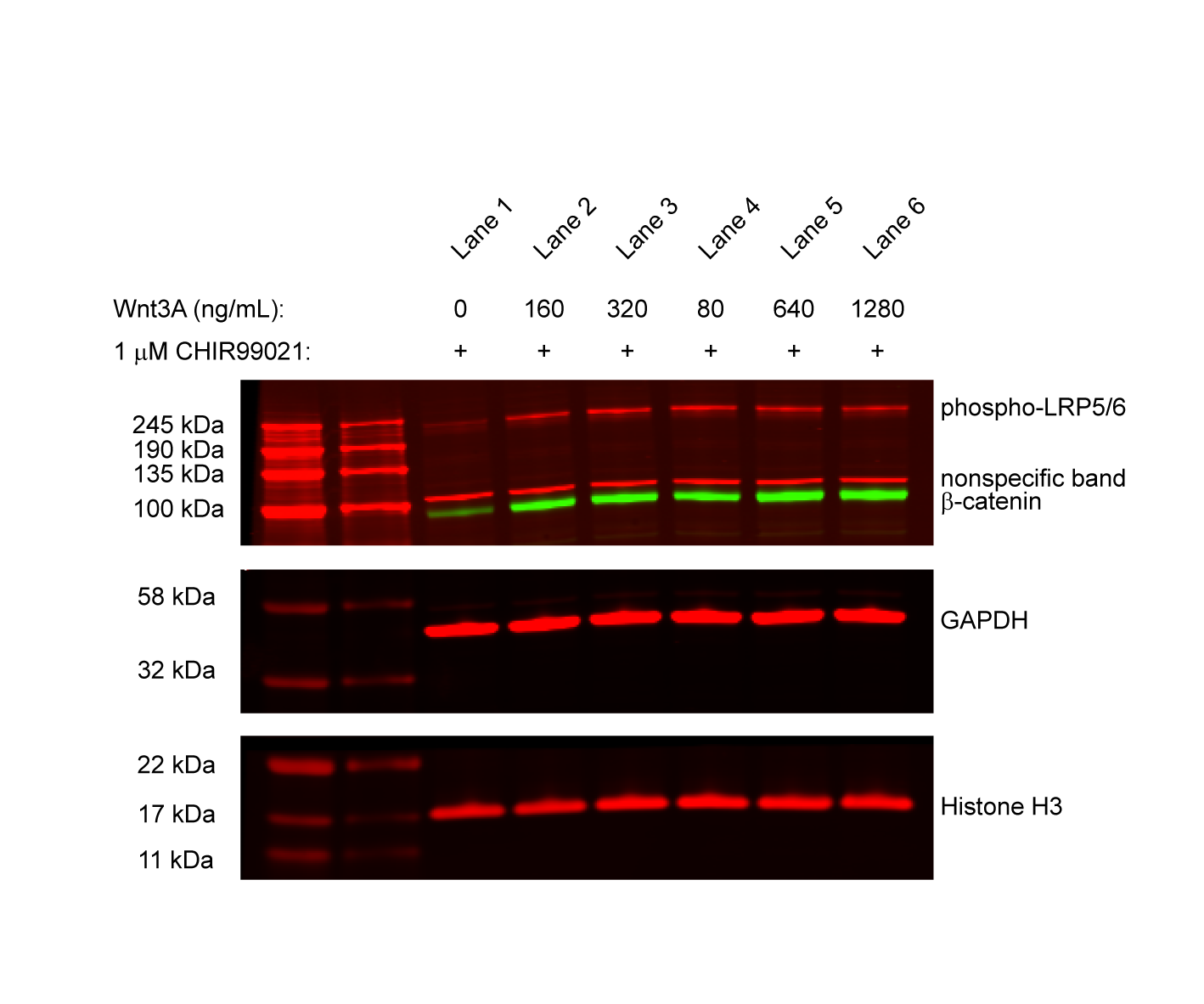

Supplement: Figure 3—source data 1. [file elife-33617-fig3-data1.zip › Figure 3 Source Data 1/Figure 3C Gel Images/Figure 3C - Gel 8-01.tif]

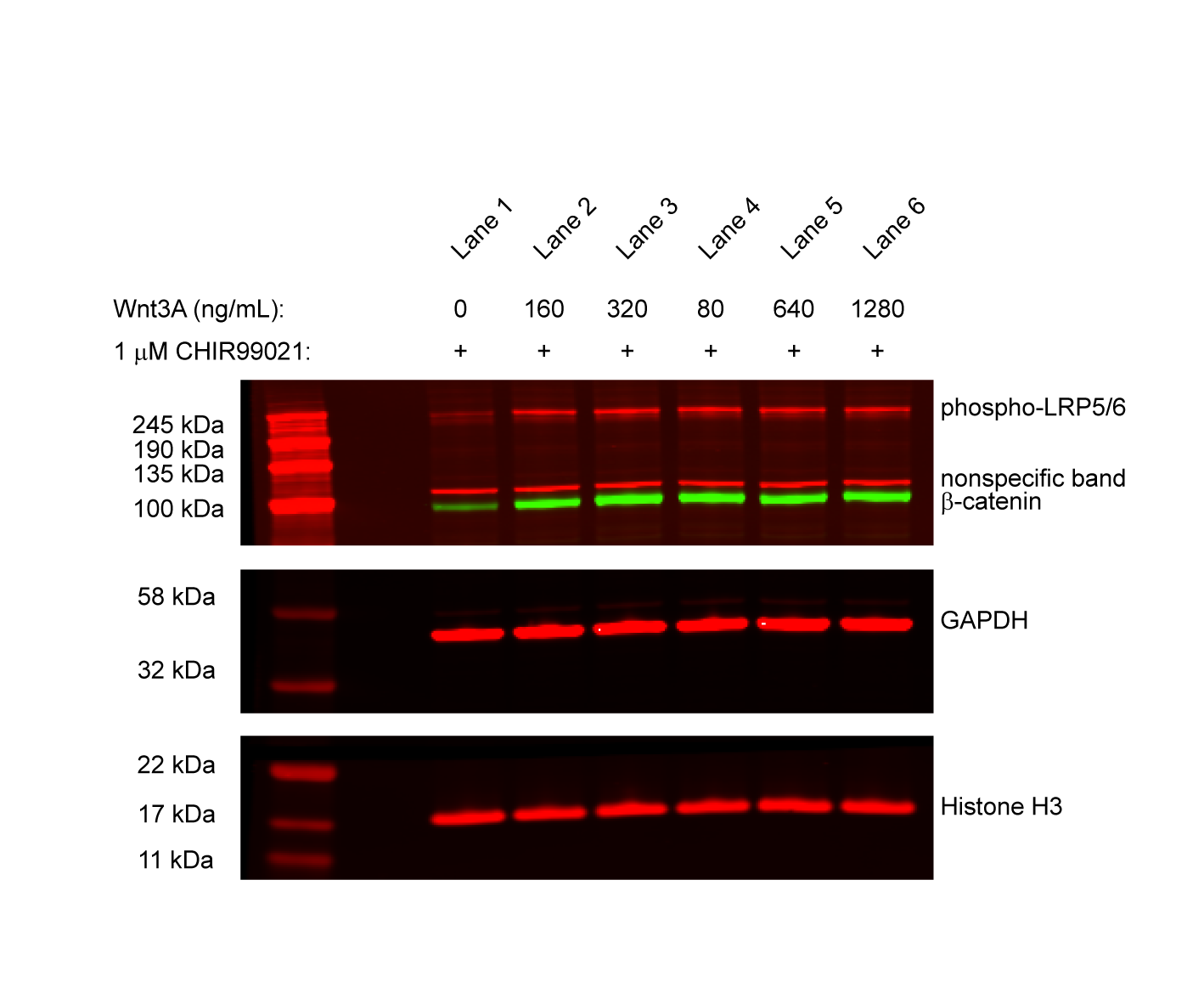

Supplement: Figure 3—source data 1. [file elife-33617-fig3-data1.zip › Figure 3 Source Data 1/Figure 3C Gel Images/Figure 3C - Gel 9-01.tif]

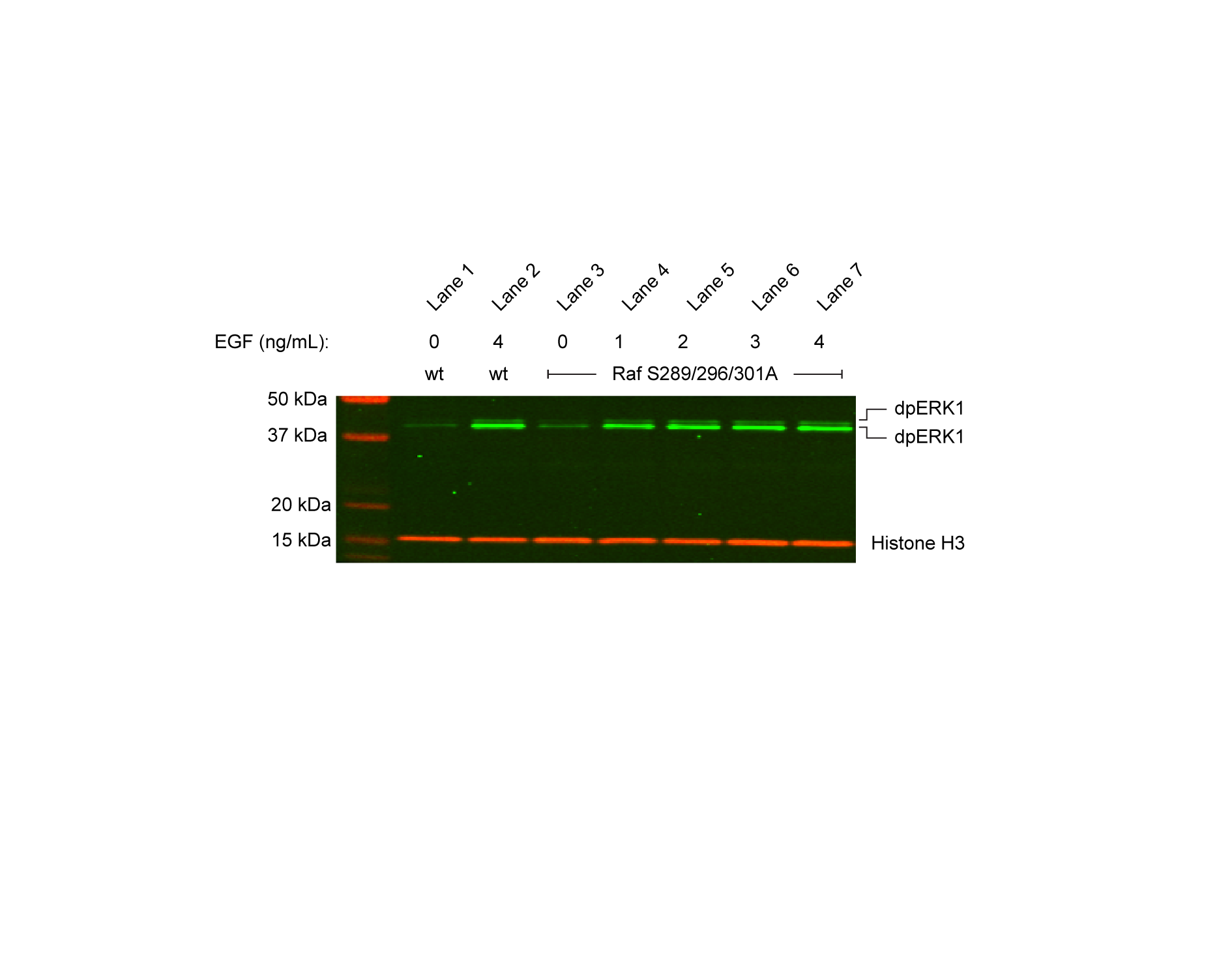

Supplement: Figure 3—source data 1. [file elife-33617-fig3-data1.zip › Figure 3 Source Data 1/Figure 3D Gel Images/Figure 3D - Gel 1-01.tif]

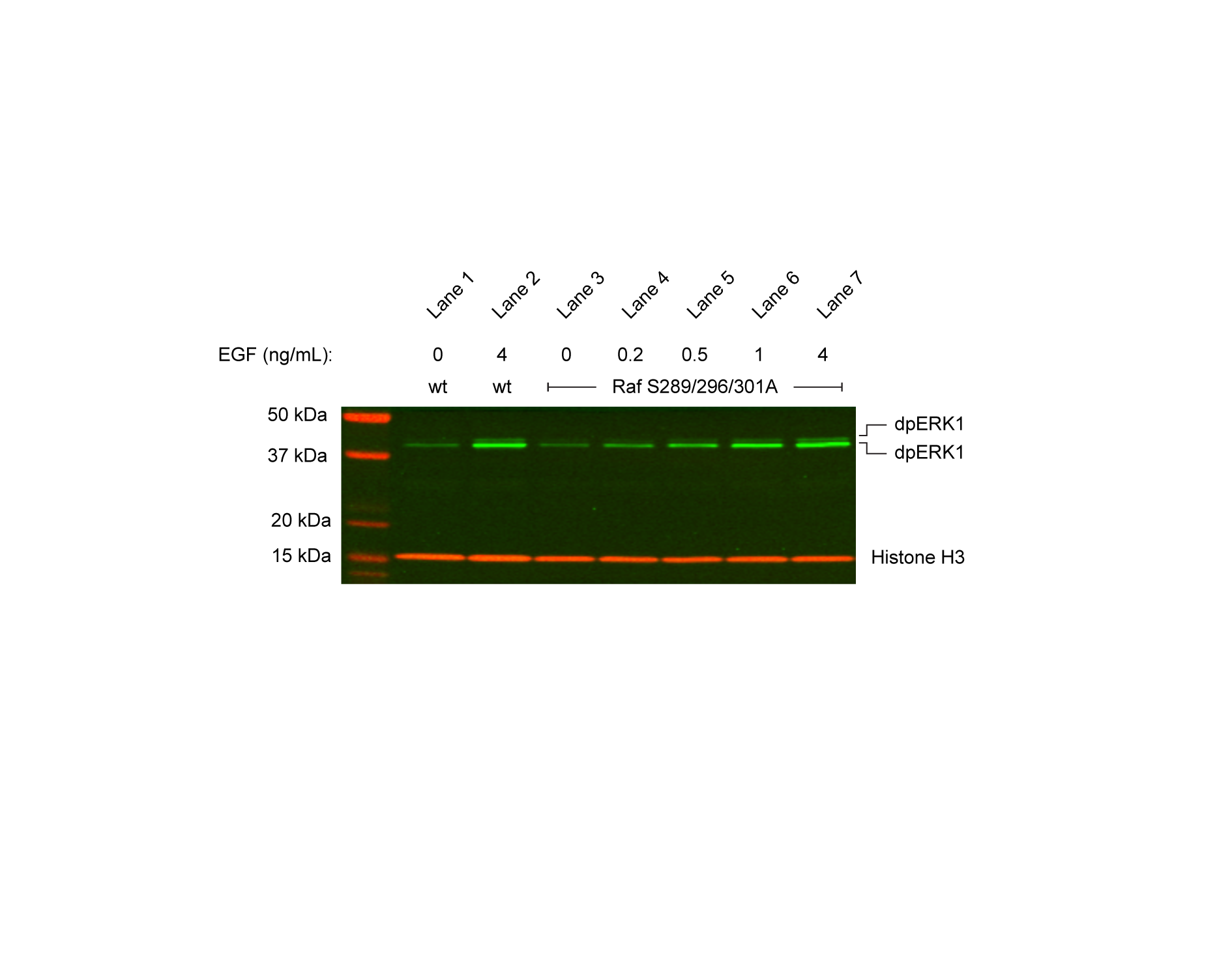

Supplement: Figure 3—source data 1. [file elife-33617-fig3-data1.zip › Figure 3 Source Data 1/Figure 3D Gel Images/Figure 3D - Gel 2-01.tif]

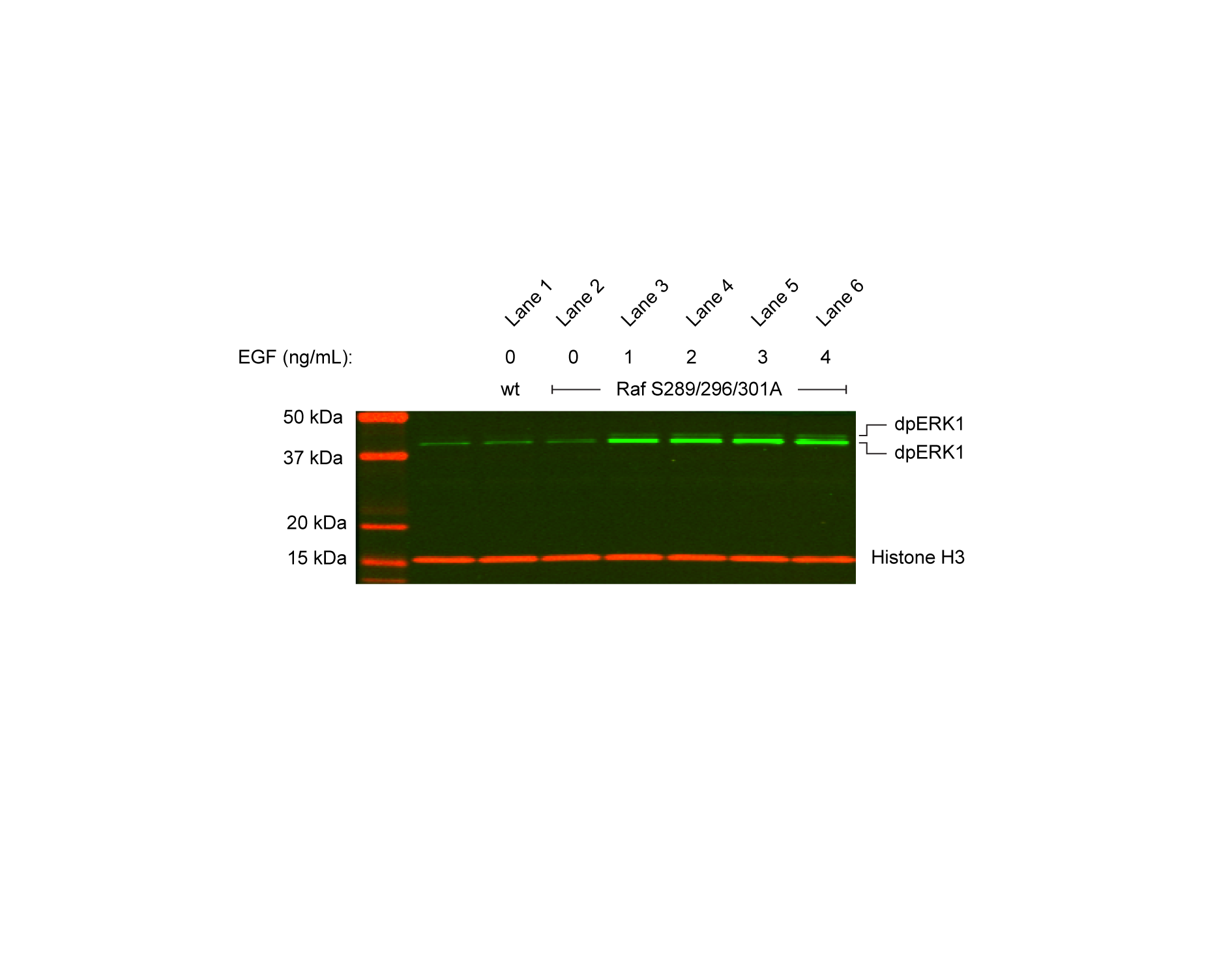

Supplement: Figure 3—source data 1. [file elife-33617-fig3-data1.zip › Figure 3 Source Data 1/Figure 3D Gel Images/Figure 3D - Gel 3-01.tif]
